# High-Intensity Interval Training as an Efficacious Alternative to Moderate-Intensity Continuous Training for Adults with Prediabetes

**DOI:** 10.1155/2015/191595

**Published:** 2015-03-30

**Authors:** Mary E. Jung, Jessica E. Bourne, Mark R. Beauchamp, Emily Robinson, Jonathan P. Little

**Affiliations:** ^1^School of Health and Exercise Sciences, University of British Columbia, Okanagan Campus, 3333 University Way, Kelowna, BC, Canada V1V 1V7; ^2^School of Kinesiology, University of British Columbia, 210-6081 University Boulevard, Vancouver, BC, Canada V6T 1Z1

## Abstract

*Aims*. High-intensity interval training (HIIT) leads to improvements in various markers of cardiometabolic health but adherence to HIIT following a supervised laboratory intervention has yet to be tested. We compared self-report and objective measures of physical activity after one month of independent exercise in individuals with prediabetes who were randomized to HIIT (*n* = 15) or traditional moderate-intensity continuous training (MICT, *n* = 17). *Method*. After completing 10 sessions of supervised training participants were asked to perform HIIT or MICT three times per week for four weeks. *Results*. Individuals in HIIT (89 ± 11%) adhered to their prescribed protocol to a greater extent than individuals in MICT (71 ± 31%) as determined by training logs completed over one-month follow-up (*P* = 0.05, Cohen's *d* = 0.75). Minutes spent in vigorous physical activity per week measured by accelerometer were higher in HIIT (24 ± 18) as compared to MICT (11 ± 10) at one-month follow-up (*P* = 0.049, Cohen's *d* = 0.92). Cardiorespiratory fitness and systolic blood pressure assessed at one-month follow-up were equally improved (*P*'s < 0.05). *Conclusions*. This study provides preliminary evidence that individuals with prediabetes can adhere to HIIT over the short-term and do so at a level that is greater than MICT.

## 1. Introduction

It is estimated that ~35% of all US adults have prediabetes and are therefore at high risk for future development of type 2 diabetes (T2D) and cardiovascular disease (CVD, [1]). Regular exercise can help prevent the progression of prediabetes to T2D [2]. Landmark trials, including the Diabetes Prevention Program, have shown that a lifestyle intervention including 150 minutes of moderate-intensity physical activity (primarily walking) per week can reduce the incidence of T2D by ~58% [3]. These findings have contributed to the development of physical activity guidelines in several countries, which typically recommend at least 150 minutes per week of moderate-intensity activity to improve health [4–6]. Unfortunately, the vast majority of individuals fail to achieve this target. Specifically, large population-based studies in the US [7], UK [8], and Canada [9] that objectively measure physical activity by accelerometer suggest that only 15–20% of adults accumulate 150 minutes of moderate-intensity physical activity per week. Alternative forms of physical activity that can increase exercise adherence may therefore be attractive for the prevention of T2D.

High-intensity interval training (HIIT) involves brief bursts of vigorous exercise separated by periods of rest or recovery. HIIT has garnered attention in recent years because it promotes cardiometabolic adaptations that are often superior to moderate-intensity continuous training (MICT) across a range of clinical populations [10, 11]. The benefits of HIIT are intriguing because adaptations to HIIT appear to occur with considerably less exercise time commitment than traditional exercise guidelines [12]. For example, time-efficient HIIT has been shown to rapidly improve glucose control in individuals with prediabetes [13, 14] and T2D [12, 15]. Despite the evidence for promising health benefits in individuals with prediabetes (for review see [16, 17]) and suggestions that HIIT may represent a time-efficient health promoting exercise strategy [18], there are no studies that have assessed adherence to HIIT in individuals at high risk for developing T2D.

Epidemiological data suggests that more vigorous-intensity physical activity may confer greater benefits to metabolic health [19]. In support of the efficacy of high-intensity exercise, the American College of Sports Medicine and American Heart Association guidelines for maintaining fitness and health recommend 150 minutes of moderate or 75 minutes of vigorous physical activity for optimal health. These recommendations imply that equivalent health benefits are achieved in less time, provided the intensity of exercise is high. Advocating this lower volume of vigorous physical activity is of potential importance for exercise adherence as “lack of time” is the most commonly cited perceived barrier to regular exercise participation [20]. However, suggestions of prescribing vigorous-intensity physical activity for health promotion and T2D prevention have been met with reluctance in the scientific, medical, and lay communities, as it is presumed to be too intense and aversive (and thus less likely to be adhered to) for individuals who are sedentary and/or at increased risk of chronic disease [21, 22]. By virtue of the built-in rest periods and break in monotony, HIIT is different than high-intensity continuous exercise and may represent an alternative and more feasible option for introducing vigorous activity for health promotion in individuals with prediabetes. In fact, we have recently shown that a majority of inactive adults (*n* = 44) prefer HIIT (62%) to MICT (20%) and high-intensity continuous training (3%) after experiencing a single bout of each type of exercise [23].

The objective of this feasibility study was to determine the utility of HIIT as an exercise strategy for promoting short-term exercise adherence in comparison to traditional MICT. Individuals with prediabetes were randomized to a two-week supervised exercise intervention involving either HIIT or MICT. After the supervised phase, they were prescribed to maintain, on their own, thrice-weekly exercise sessions of their respective modality. The primary outcome was exercise adherence assessed by accelerometer and training logs after one month of independent exercise. As this preliminary pilot study was the first to examine independent exercise adherence to HIIT in comparison to traditionally prescribed MICT, the time point of one month was chosen a priori because it was deemed important to ascertain the feasibility of prescribing HIIT prior to conducting longer-term adherence trials. A secondary objective was to compare fitness and anthropometric changes in response to HIIT or MICT to determine which type of exercise might lead to greater cardiometabolic benefits. We hypothesized that HIIT would lead to greater adherence and improvements in cardiometabolic risk when compared to MICT.

## 2. Materials and Methods

### 2.1. Participants

**Figure 1:**
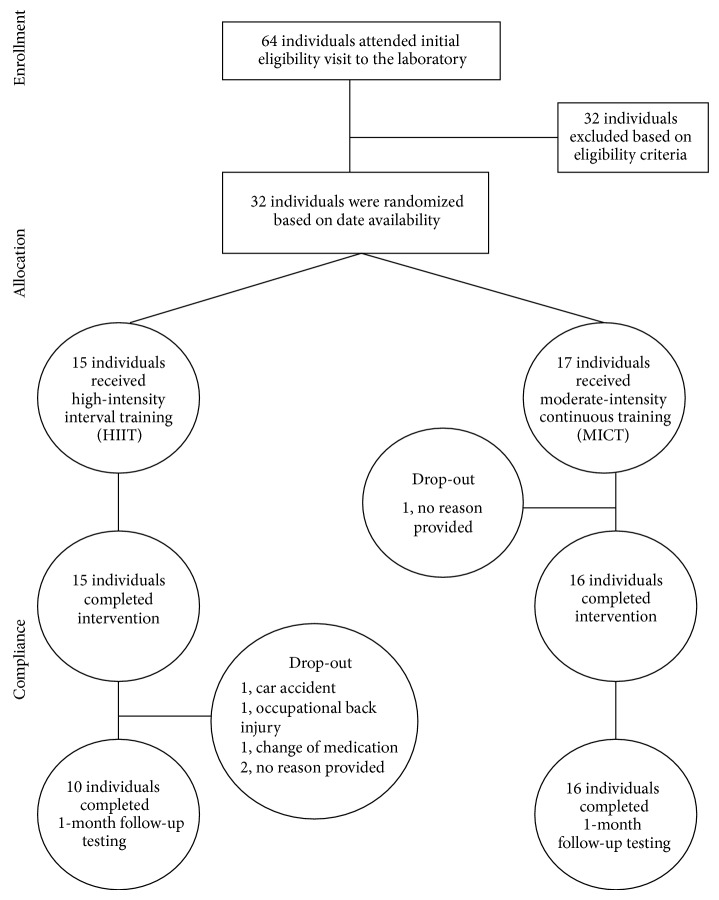
Flow of participants through the intervention.

Participants with prediabetes between the ages of 30 and 60 years were recruited from posters, online message boards, and word of mouth. After expressing interest, a phone interview was conducted to assess preliminary eligibility. Participants were considered to have prediabetes based on one of the following criteria: (1) physician-diagnosed, (2) HbA1c values between 5.7 and 6.4% (American Diabetes Association [24]) assessed using a clinically validated point-of-care monitor (HbA1c Now, Bayer Inc., Ontario, Canada), (3) fasting blood glucose of 5.6–6.9 mmol/L [24], and/or (4) a CANRISK questionnaire score of moderate/high (>21 [25]). To be eligible, participants also had to be inactive based on completion of less than two 30-minute bouts of moderate-intensity physical activity per week over the past six months. Participants completed the Canadian Society for Exercise Physiology (CSEP) Physical Activity Readiness Questionnaire-Plus (PAR-Q+ [26]) and were cleared for participation in vigorous activity by a CSEP Certified Exercise Physiologist. Exclusion criteria included diagnosed diabetes, glucose lowering medications, uncontrolled hypertension (blood pressure > 160/90), history of heart disease, previous myocardial infarction or stroke, and any contraindications to exercise. Thirty-two participants met the eligibility criteria and were enrolled in the study after providing written informed consent. Flow of participants is depicted in Figure 1.

### 2.2. Procedures

The study design was approved by the Institutional Clinical Research Ethics Board. Following baseline testing (including accelerometry, cardiorespiratory fitness, blood pressure, and anthropometrics), eligible participants were randomized to HIIT or MICT conditions involving ten sessions of exercise performed over a 12-day period (i.e., Monday-Friday over two weeks with Saturday and Sunday as rest days). Exercise prescriptions for each condition were progressive in nature and were designed to be matched for external work [13]. Specifically, individuals randomized to HIIT began with four intervals lasting 1 minute each at an intensity that elicited ~90% peak heart rate (HRpeak) separated by 1-minute of low intensity recovery and increased to 10 × 1 min intervals by day 10, which was based on previously published studies in individuals with, and at risk for, T2D [12, 13, 15]. A 3-minute warm-up and 2-minute cool-down was incorporated into the HIIT sessions. Individuals randomized to MICT began with 20 minutes of continuous activity at ~65% HRpeak and gradually increased duration to 50 minutes by day 10. Participants self-selected exercise modality (walking outdoors, elliptical machine, treadmill walking, or stationary cycling) for each bout to encourage autonomy. Two trained research assistants (RAs) supervised participants during the training phase. To decrease reliance on staff and encourage the practice of independent exercise, participants completed 3 of the 10 training days (days 4, 7, and 9) at home unsupervised. Participants wore a heart rate monitor during each exercise session (supervised and unsupervised) to monitor exercise intensity and ensure compliance. In addition, participants recorded their exercise bouts in a logbook on both lab and home training days.

Participants in both groups received 10 minutes of behavioural counselling each day they trained in the lab (for a total of 70 minutes). The aim of this one-on-one counselling was to further prepare participants for transition from lab-based training to independent exercise. The behavioural counselling was grounded in social cognitive theory [27] and identical in content for both conditions. Topics covered during counselling included overcoming exercise barriers, bolstering self-regulatory self-efficacy, planning, and increasing awareness for the physical and psychological benefits of exercise. Taken together, the short-term intervention served to introduce participants to their assigned exercise modality (HIIT or MICT) while providing evidence-based strategies and skills in attempts to bolster continual exercise adherence.

During the supervised training phase, average HRpeak during HIIT (including rest intervals, warm-up, and cool-down) was 82 ± 3% HRpeak, confirming that HIIT sessions were in the vigorous domain. Average HRpeak during MICT was 67 ± 5%, confirming that MICT sessions were in the moderate domain. Following the supervised training phase, participants were instructed to maintain HIIT or MICT three days per week independently. Specifically, individuals randomized to HIIT were prescribed three exercise sessions per week involving 10 × 1-minute intervals at an interval intensity of ~90% HRpeak separated by 1 minute of easy recovery with a 3-minute warm-up and 2-minute cool down (for a total of 25 minutes of vigorous exercise), while participants randomized to MICT were prescribed three sessions per week of 50-minute continuous exercise at an intensity of ~65% HRpeak. Therefore, the HIIT group was prescribed vigorous exercise requiring 75 minutes of time commitment per week whereas the MICT group was prescribed 150 minutes of moderate activity per week. Training logs were provided and participants were instructed to estimate exercise intensity based on physiological cues and ratings of perceived exertion (RPE) taught during supervised training days. Accelerometers were used to objectively measure physical activity over seven days after four weeks of independent exercise. Adherence was assessed based on accelerometers and training logs.

### 2.3. Measures

#### 2.3.1. Heart Rate

During the supervised training intervention, heart rate was recorded using downloadable Polar heart rate monitors (Polar FT7, Finland) to ensure that participants were working at the prescribed exercise intensity.

#### 2.3.2. Training Logbooks

Participants were provided with a training logbook to complete during the intervention as well as during the subsequent four weeks of independent exercise. Participants were asked to record (a) the activity performed, (b) the minutes spent engaging in the activity, (c) the number of intervals conducted (for the HIIT condition exclusively), and (d) how hard the session was (RPE, CR-10 scale, [28]) for the session. Training logbooks were returned at the four-week follow-up and were analyzed by calculating the percentage adherence (i.e., number of exercise sessions divided by the number of prescribed sessions times 100% [29]) and average RPE.

#### 2.3.3. Accelerometers

The Actigraph GT1M accelerometer (Actigraph, LCC, Fort Walton Beach, FL, USA) was used to objectively measure physical activity. This device is a dual-axis motion sensor that records vertical and horizontal accelerations allowing researchers to identify time spent in various physical activity intensities on a daily basis. Participants were instructed to wear the accelerometer for all waking hours of the day and to only remove it for sleeping or water-based activities. The monitor was worn on, or just above, their right hip for seven consecutive days prior to the intervention (baseline) and after 4 weeks of independent exercise. Accelerometer data were also collected during the intervention (days 7, 8, and 9) as a manipulation check. Each time participants wore an accelerometer, they were also asked to monitor their wear time with a self-report log.

Epoch lengths were specified at 5 seconds and were summed as counts per minute. Valid wear time was ascertained using Choi and colleagues [30] parameters (i.e., nonwear is classified as 90 minutes of consecutive zeros, allowing for nonzero counts up to 2 minutes, if no counts are detected during the 30-minute counts before and after this interval). A trained researcher verified valid wear time using the raw data and self-report logs. Freedson et al. [31] cut-points were used to identify time spent in moderate (≥1,952 counts/min), vigorous (≥5,725 counts/min), and moderate-to-vigorous physical activity (MVPA, sum of moderate and vigorous) during wear time on valid days (i.e., ≥10 hours of wear time per day, without any excessive counts ≥20,000 counts/min). Purposeful exercise was operationalized as minutes spent in MVPA in bouts of at least 10 minutes (MVPA10+ [31]) based on physical activity guidelines, which specify bouts, should be accumulated in bouts of 10 minutes or more. MVPA10+ is a more appropriate measure of purposeful exercise [32] and therefore allows a more direct objective assessment of exercise adherence. Participants were required to have at least 3 valid days to be included in the analyses at baseline and one month [33]. Time spent in moderate activity and vigorous activity, total MVPA, and MVPA10+ were calculated for each valid day independently. Time spent in the various intensities was averaged across valid wear days and multiplied by seven to provide a weekly estimate of physical activity at baseline and one-month follow-up.

#### 2.3.4. Cardiorespiratory Fitness

Participants performed a continuous incremental ramp maximal exercise test on an electronically braked cycle ergometer (Lode Excalibur, Netherlands) before intervention and at one-month follow-up to determine peak oxygen uptake (VO_2peak_), HRpeak, and peak power output (*W*_peak_). Expired gas was collected continuously by a metabolic cart (Parvomedics TrueOne 2400, Salt Lake City, Utah, USA) that was calibrated with gases of known concentration and a 3.0 L syringe prior to every test. The test started at 50 Watts and increased by 15 Watts/min. Verbal encouragement was provided to participants throughout the test, which was terminated upon volitional exhaustion or when revolutions per minute fell below 50. VO_2peak_ was defined as the highest 30 sec average for VO_2_ (in L/min and mL/kg/min). Criteria for achieving VO_2peak_ were (i) respiratory exchange ratio >1.15; (ii) plateau in VO_2_; (iii) reaching age-predicted HRpeak (220-age); and/or (iv) volitional exhaustion.

#### 2.3.5. Anthropometrics and Blood Pressure

Body mass, height (SECA, 700 SECA, Hamburg, Germany), waist circumference (WC, measured at the level of the umbilicus [34]), and blood pressure (BP [35]) were measured in the morning after an overnight fast at baseline testing and one-month follow-up using standard procedures.

#### 2.3.6. Analytical Plan

Data were analyzed using SPSS Statistics (v22, 2013) on a per-protocol basis. Independent samples *t*-tests were conducted to examine differences between the two exercise conditions on (a) percentage adherence to the exercise prescription and (b) total number of minutes of purposeful physical activity, reported over the four weeks of independent exercise in the training logbooks. Analyses of covariance (ANCOVA) were conducted to examine differences in accelerometer-derived moderate physical activity, vigorous physical activity, total MVPA, and MVPA10+ between the conditions during the fifth week of independent exercise, after controlling for baseline measures as covariates. In order to examine changes in cardiorespiratory fitness, anthropometry, and blood pressure between baseline and one-month follow-up, a series of two-way (group × time) repeated measures ANOVA were conducted. Independent sample *t*-tests were conducted prior to running the repeated measures ANOVA to ensure there were no differences in baseline activity levels or fitness data between conditions. Significance was set at *P* ≤ 0.05.

## 3. Results

All 32 participants (17 in MICT, 15 in HIIT) completed the short-term supervised intervention with no complications (see Figure 1 for participation flow through the study). Descriptive statistics for these individuals are provided in Table 1. One participant in MICT did not return for one-month testing and was deemed lost to follow-up as she could not be reached after repeated attempts to contact. The remaining 16 MICT participants returned for 1-month follow-up. In the HIIT condition, all 15 participants completed the 2-week intervention. At one-month follow-up, two participants were deemed lost to follow-up and did not provide specific reasons. Three participants were unable to complete one-month follow-up for reasons unrelated to the study (car accident, work-related injury, diagnosis of depression, and change in medication). Therefore, a total of 16 and 10 participants completed one-month follow-up for MICT and HIIT, respectively. Of the individuals that completed one-month follow-up, accelerometer data were available for 10 individuals in the HIIT condition and 13 individuals in the MICT condition (two participants did not accrue the minimum of valid wear days and one accelerometer malfunctioned displaying counts >20,000 during wear time). Mean (±SD) daily wear time was 855 (±175) minutes at baseline and 846 (±63) minutes at follow-up. Eighty-six percent and 98% of participants provided at least 5 valid wear days at baseline and one-month follow-up, respectively.

### 3.1. Manipulation Check

**Figure 2:**
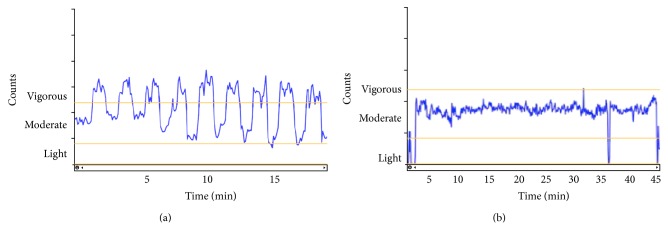
(a) Representative accelerometer counts recorded throughout a bout of HIIT during the intervention. Note: nine intervals were completed in this training session. (b) Representative accelerometer counts recorded throughout a bout of MICT during the intervention. Note: approximately 45 minutes of MICT was completed in this training session.

Accelerometer data collected during the intervention revealed that the accelerometers were able to clearly detect intermittent patterns of HIIT in comparison to the continuous moderate activity characteristic of MICT (see Figures 2(a) and 2(b)). Visual inspection of the accelerometer data (days 7 and 9) and confirmation of exercise heart rate data (days 4, 7, and 9) revealed that all participants were working at the prescribed exercise intensity during the unsupervised training days.

### 3.2. Self-Report Physical Activity Behaviour

To examine differences in percentage of overall adherence to the exercise prescription over one-month follow-up, an independent *t*-test was conducted. Results revealed a significant difference in adherence rates between HIIT and MICT, *t*(18.96) = 2.08, *P* = 0.05, Cohen's *d* = 0.75. Specifically, individuals in the HIIT condition (89 ± 11%) adhered to their prescribed protocol, to a greater extent than individuals in the MICT condition (71 ± 31%). The same analysis revealed a significant difference in average ratings of perceived exertion between the two conditions, *t*(23) = 2.80, *P* = 0.01, Cohen's *d* = 1.19. As expected, HIIT was perceived to be of greater intensity than MICT (HIIT = 7.4 ± 1.7, MICT = 5.4 ± 1.8).

### 3.3. Objective Physical Activity Behaviour

A series of ANCOVA revealed no significant differences between the HIIT and MICT conditions in the average number of minutes spent in (a) purposeful bouts of MVPA per week (i.e., MVPA10+), (b) overall MVPA per week, and (c) moderate activity per week (*P*'s > 0.05). Means and standard deviations for each condition are displayed in Table 2. There was, however, a significant difference between the average number of minutes spent in vigorous physical activity per week between the two conditions, *F*(1, 20) = 4.41, *P* = 0.049, Cohen's *d* = 0.92, with HIIT being greater than MICT.

### 3.4. Changes in Fitness Parameters

Changes in fitness and anthropometric measures from baseline to one-month follow-up for HIIT and MICT are shown in Table 3. Repeated measures ANOVA revealed a significant main effect of time for absolute VO_2peak_, *F*(1, 24) = 20.96, *P* < 0.001, Cohen's *d* = 1.92; relative VO_2peak_, *F*(1, 24) = 14.82, *P* < 0.001, Cohen's *d* = 1.62; and peak power output, *F*(1, 24) = 14.08, *P* < 0.001, Cohen's *d* = 1.57, indicating an increase in cardiorespiratory fitness assessed at one-month follow-up. There were no main effects for group or group by time interactions for these fitness parameter variables, indicating that there were no differences in the degree of change experienced between HIIT and MICT over time.

Maximal heart rate achieved at the end of the graded exercise test, body mass, and waist circumference did not change significantly from baseline to one-month follow-up in either HIIT or MICT (*P*'s > 0.05). There was a significant main effect of time for systolic blood pressure, *F*(1, 24) = 17.36, *P* < 0.001, Cohen's *d* = 1.75, such that it decreased at one-month follow-up for both conditions. Diastolic blood pressure tended to be lower at one-month follow-up; however, this main effect for time did not reach statistical significance (*P* = 0.11, Cohen's *d* = 0.13).

## 4. Discussion

Physical activity is key in the prevention of T2D and cardiovascular disease in individuals with prediabetes; yet exercise adherence is low in this population [36]. HIIT has recently been touted as an effective and time-efficient exercise option for improving cardiometabolic health [18, 37]. However, adherence to HIIT outside of a supervised lab-based intervention has not been tested. The present study demonstrates that individuals with prediabetes can adhere to HIIT* independently* for one month following a very brief supervised laboratory intervention. Interestingly, adherence to HIIT, assessed by self-report in free-living conditions, was greater than standard care exercise involving MICT. In addition, only those individuals randomized to HIIT increased their vigorous physical activity (>6 METS) from baseline to one-month after intervention.

Current physical activity guidelines recognize that vigorous physical activity can elicit health benefits in less time when compared to moderate-intensity physical activity. Indeed, guidelines imply that 75 minutes of vigorous activity is equivalent to 150 minutes of moderate activity [38]. However, it is generally assumed that adherence to vigorous exercise will be lower than moderate exercise. This appears based on studies examining the affective response to acute bouts of exercise at different intensities [39]. Our findings provide initial evidence that short-term adherence to vigorous exercise performed as HIIT may be superior to traditional moderate-intensity continuous exercise.

In addition to time-efficiency, vigorous exercise may elicit benefits over and above those accrued through moderate-intensity activity. Specifically, NHANES data suggests that individuals accumulating more vigorous-intensity physical activity have reduced odds of metabolic syndrome (closely related to prediabetes), independent of total physical activity levels [19]. Further, when matched for energy expenditure, vigorous physical activity had a greater influence on metabolic syndrome than did moderate intensity physical activity. In other words, it appears that vigorous-intensity physical activity has an important independent role in the prevention of cardiometabolic disease. By design, only those randomized to HIIT in this feasibility study would be expected to increase their minutes of vigorous-intensity physical activity. Indeed, those randomized to HIIT increased their vigorous-intensity physical activity by ~12 minutes per week while individuals randomized to MICT in this study experienced no increase.

There were no significant differences between conditions in minutes spent in moderate-intensity physical activity or MVPA10+ assessed at one-month follow-up. This was despite individuals in MICT being prescribed 150 minutes of moderate-intensity physical activity per week as compared to individuals in HIIT being prescribed 75 minutes of intermittent vigorous-intensity physical activity. This suggests that objectively measured moderate and total physical activity increased to a similar extent in both groups despite those in the HIIT group being prescribed only ~50% the amount of time to exercise as those in MICT. Taken together, individuals in HIIT effectively increased time spent in vigorous-intensity physical activity and also saw increases in their moderate-intensity physical activity that were comparable to individuals in MICT.

Assessing levels of physical activity objectively by accelerometer following supervised training interventions remains largely unexplored. Our findings indicate that physical activity levels can be increased for up to one month following a brief (10-day) exercise intervention as measured by accelerometry. Specifically, time spent in MVPA10+ was increased following both HIIT and MICT. This was further confirmed with the training logbook data collected in our sample, with >70% adherence measured in both groups. Both HIIT and MICT increased VO_2peak_ assessed at one-month follow-up, indicating an improvement in fitness. This is promising considering all participants were training under their own volition for one month. A recent meta-analyses reported that the increase in VO_2peak_ following 12–16 weeks of supervised HIIT is approximately double the increase seen following MICT [17]. Thus, the increase in adherence and vigorous physical activity in the HIIT group may, if adhered to for longer periods of time, lead to greater reductions in cardiometabolic risk when compared to MICT.

The present feasibility study is the first of its kind to assess adherence to HIIT following a supervised laboratory intervention. Such research is needed to determine whether HIIT is a viable health-enhancing exercise strategy in the real world. However, our study is not without limitations. First, we examined independent exercise over a short time period. The results are promising but certainly more research is needed to determine whether individuals with prediabetes can adhere to HIIT over the long term. Second, our sample size was small and larger studies are warranted to confirm and expand our preliminary findings. Loss to follow-up was similar in HIIT (2 participants) versus MICT (1 participant), although we were limited by the fact that a further 3 participants in the HIIT group did not complete one-month follow-up testing due to factors unrelated to the study protocol. There are natural safety concerns when implementing vigorous exercise. All participants in our study completed two weeks of supervised HIIT without any reported complications but our study was not designed to examine safety and musculoskeletal injuries in response to HIIT compared to traditional MICT. The small sample size of the current investigation does not permit an accurate assessment of the safety or injury risk potential of HIIT or MICT.

## 5. Conclusion

Adherence to exercise in individuals at risk for T2D is remarkably low. HIIT has recently gained popularity as a potential health-enhancing exercise strategy that is time-efficient and distinct from traditional MICT [17, 18]. However, the application of HIIT for persons at risk of chronic disease has been questioned due to perceptions that adherence to vigorous-intensity physical activity is unlikely. In this feasibility study, we provide preliminary evidence that individuals with prediabetes can adhere to HIIT over the short term and do so at a level that is greater than MICT. These findings support the potential utility of HIIT as an alternative exercise strategy that could bolster exercise adherence. Future studies are warranted to assess long-term adherence, cardiometabolic benefits, and safety of HIIT in individuals with prediabetes.

## Figures and Tables

**Table 1:** Baseline characteristics of study participants randomized to high-intensity interval training (HIIT, *n* = 15) and moderate-intensity continuous exercise (MICT, *n *= 17) and comparisons between conditions.

Variables	All (*n* = 32)	HIT (*n* = 15)	MICT (*n* = 17)	*P*
Age (years)	51 (10)	51 (11)	51 (10)	0.972
Gender, *n* (%)				
Male	5 (16.6%)	4 (27.7%)	1 (5.8%)	
Female	27 (84.4%)	11 (73.3%)	16 (94.1%)	
Body mass (kg)	89.9 (18.8)	89.8 (23)	90 (15)	0.985
Body mass index (kg/m^2^)	32.9 (6.3)	33.1 (7.7)	32.8 (5)	0.904
Waist circumference (cm)	107.3 (14.2)	105.2 (16.5)	109.3 (12.1)	0.426
VO_2_ absolute (L/min)	1.81 (0.44)	1.76 (0.38)	1.85 (0.49)	0.551
VO_2_ relative (mL/kg/min)	20.23 (4.48)	19.99 (3.58)	20.44 (5.25)	0.783
CANRISK	32 (11)	29 (10)	34 (11)	0.121
HbA1c (%)	5.7 (0.53)	6.1 (0.60)	5.5 (0.36)	0.077
Ethnic origin, *n* (%)				
Caucasian	30 (93.8%)	14 (93.3%)	16 (94.1%)	
Latin American	1 (3.1%)	0	1 (5.9%)	
European	1 (3.1%)	1 (6.7%)	0	
Annual income, *n* (%)				
$0–$24,999	4 (12.5%)	0	4 (23.5%)	
$25,000–$49,999	6 (18.8%)	3 (20.0%)	3 (17.6%)	
$50,000–$74,999	10 (31.3%)	5 (33.3%)	5 (29.4%)	
$75,000–$99,999	7 (21.9%)	3 (20.0%)	4 (23.5%)	
$100,000+	5 (15.7%)	4 (26.7%)	1 (5.9%)	

All values are mean (SD) unless indicated as *n* (%).

**Table 2:** Mean values for physical activity behaviour before the intervention (pre) and at one-month follow-up (post) for HIIT and MICT, as measured by accelerometers.

Variable	HIIT (*n* = 10)		MICT (*n* = 13)	
	Mean number of minutes per week (SD)			
	Pre	1-month follow-up	Pre	1-month follow-up
MVPA10+	24.6 (25.6)	51.6^a^ (41.1)	39.6 (54.3)	84.1^a^ (90.0)
MVPA	287.7 (112.5)	309.4 (70.4)	311.3 (142.6)	312.5 (96.5)
Moderate activity	277.0 (103.6)	285.8 (60.4)	302.2 (137.8)	301.8 (94.6)
Vigorous activity	11.6 (10.7)	23.7^∗a^ (18.0)	9.1 (8.0)	10.7^a^ (9.7)

^∗^Difference between HIIT and MICT, *P* < 0.05.

^a^Main effect of time, *P* < 0.05.

**Table 3:** Changes in fitness and cardiometabolic parameters (mean [SD]) before and 1 month following intervention for study participants randomized to high-intensity interval training (HIIT, *n* = 10) and moderate-intensity continuous exercise (MICT, *n* = 16) and overall (*n* = 26) effect of time. The current results represent per-protocol analysis.

	HIIT		MICT		All		*P* ^∗^
	Pre	1 month	Pre	1 month	Pre	1 month	
Body mass (Kg)	79.0 (17.2)	79.3 (16.4)	88.2 (13.4)	89.3 (12.6)	84.9 (15.3)	85.5 (14.8)	0.70
BMI	29.8 (5.5)	29.9 (5.1)	32.1 (4.1)	31.9 (3.7)	31.2 (4.7)	31.2 (4.3)	0.81
Waist circumference (cm)	97.5 (12.4)	97.5 (13)	108.1 (11.5)	107.4 (10.2)	104.1 (12.8)	103.6 (12.1)	0.65
Blood pressure (mmHg)							
Systolic	133 (21)	125 (15)	131 (8)	124 (7)	132 (14)	124 (10)	<0.001
Diastolic	82 (7)	79 (8)	83 (8)	81 (8)	82 (8)	81 (8)	0.11
Absolute VO_2peak_ (L/min)	1.64 (0.38)	1.79 (0.49)	1.86 (0.51)	1.96 (0.48)	1.77 (0.47)	1.89 (0.48)	<0.001
Relative VO_2peak_ (mL/kg/min)	20.71 (3.25)	22.63 (4.07)	20.79 (5.21)	21.98 (4.55)	20.76 (4.48)	22.23 (4.30)	<0.001
*W*_peak_ (Watts)	144 (30)	153 (35)	156 (39)	163 (40)	152 (36)	159 (35)	<0.001
Power (Watt/kg)	1.74 (0.47)	1.95 (0.37)	1.76 (0.40)	1.84 (0.35)	1.75 (0.42)	1.88 (0.35)	0.02
*W*_peak_ (BPM)	165 (17)	166 (15)	161 (18)	165 (12)	163 (17)	165 (13)	0.14

^∗^ *P* value reflects main effect of time from pre to 1 month as per the repeated measures ANOVA.
